# Enhanced editing of *Bifidobacterium lactis* using the endogenous Type I-G CRISPR-Cas system

**DOI:** 10.1128/aem.01839-25

**Published:** 2026-01-12

**Authors:** Ourania Raftopoulou, Kendall Malmstrom, Meichen Pan, Rodolphe Barrangou

**Affiliations:** 1 Department of Plant and Microbial Biology, North Carolina State University 6798 , Raleigh, North Carolina, USA; 2 Department of Food, Bioprocessing and Nutrition Sciences, North Carolina State University 738941 , Raleigh, North Carolina, USA; Universita degli Studi di Napoli Federico II, Portici, Italy

**Keywords:** *Bifidobacterium lactis*, CRISPR-Cas, endogenous system, editing

## Abstract

Diverse *Bifidobacterium animalis* subsp. *lactis* strains are widely used as commercial probiotics. While proof-of-concept studies have shown that some strains can be edited using several CRISPR-Cas approaches, this species remains difficult to engineer, hindering functional genomic studies to establish their molecular mode of action and enhance their probiotic functionalities. Here, we show that >95% of available *B. lactis* genomes harbor a conserved Type I-G CRISPR-Cas system, which we leverage to develop and validate a broadly applicable genome editing framework. We redesigned backbone plasmids with different replicons and antibiotic resistance markers and evaluated performance across six commercial strains for transformation efficiency. A vector carrying the pBC1 origin coupled with a chloramphenicol resistance marker improved transformation in most strains. Using synthetic CRISPR arrays with self-targeting spacers in combination with homologous editing templates, we tested multiple spacers and evaluated short (600 bp) versus long (1,000 bp) homology arms. To demonstrate applicability, we generated knockouts in three glycoside hydrolases within the Balac 1593-1601 cluster, readily cured editing plasmids in non-selective medium, and performed iterative genome editing. Growth phenotyping across carbohydrates confirmed that the GH36 α-galactosidase Balac 1601 knockout abolished melibiose and raffinose utilization, and that deletions within Balac 1596 and Balac 1593 carbohydrate hydrolases produced non-canonical phenotypes, suggestive of a modulatory role associated with shift in carbon use and compensation by other pathways. These results establish a practical toolkit for editing diverse *B. lactis* strains, unravel the genomics underlying probiotic attributes, and provide a blueprint for genome engineering in other non-model probiotic bacteria.

**IMPORTANCE:**

*Bifidobacterium animalis* subsp. *lactis* strains are prominent probiotics widely formulated in foods and dietary supplements, yet remain difficult to engineer, limiting efforts to connect genes to probiotic traits and to build strains with enhanced functions. Here, we harness the native Type I-G CRISPR Cas system to enable genome editing across commercial *B. lactis* strains by optimizing a compact plasmid backbone, testing multiple spacers to achieve efficient editing, and selecting homology arms of the appropriate length for recombination. With this framework, we generate knockouts at multiple, functionally distinct loci, demonstrating target-agnostic applicability, and we cure the CRISPR-editing vectors efficiently, enabling sequential edits. This toolkit enables systematic genotype-to-phenotype mapping in *B. lactis* and provides a practical framework for strain improvement in organisms of industrial relevance.

## INTRODUCTION

Bifidobacteria are among the earliest and most abundant colonizers of the human gut, especially in breastfed infants, and they remain key members of the adult microbiota, where they contribute to carbohydrate cross-feeding, immunomodulation, and other host health benefits (1–5). Within the genus, *Bifidobacterium animalis* subsp. *lactis* is among the most widely formulated species in foods (6) and dietary supplements commercialized as probiotics (7–9), which are defined as “live microorganisms that, when administered in adequate amounts, confer a health benefit on the host” (10). However, although diverse bifidobacteria strains in general, and several *B. lactis* strains in particular, exhibit probiotic traits, the genetic determinants of these phenotypes remain largely unknown. Progress has been constrained by relatively poor transformability due to their robust cell envelope and diverse restriction modification (R-M) systems, as well as limited availability of plasmid vectors that perform reliably in recalcitrant strains (11). Classical approaches, such as single crossover plasmid integrations (12) and transposon mutagenesis (13, 14), have enabled functional studies, but they often rely on antibiotic resistance markers, can introduce polar effects that confound phenotype interpretation, and may yield constructs with stability issues. On the other hand, inducible plasmid self-destruction can yield markerless edits but still depends on transformation and host homologous recombination, requires a strain compatible inducible system, and lacks intrinsic counterselection against the wild-type genotype (15). Although recombineering and related methods are advancing in lactic acid bacteria, *Bifidobacterium lactis* still lacks a broadly applicable, marker-minimal editing toolkit that performs reliably across strains and enables robust genotype-to-phenotype mapping for the design of advanced probiotics.

Clustered regularly interspaced short palindromic repeats (CRISPR) and CRISPR-associated sequences (Cas) are prokaryotic adaptive immune systems through which bacteria and archaea capture short DNA sequences from invading phages or plasmids as spacers, and then use CRISPR-Cas effector complexes to recognize and destroy complementary sequences during subsequent encounters (16). This interference requires a protospacer adjacent motif (PAM) next to the protospacer on the invader and proceeds only when the PAM is present, thereby enabling self versus non-self discrimination and preventing self-targeting (17, 18). This mechanism, first demonstrated genetically in *Streptococcus thermophilus*, established CRISPR as an acquired antiviral defense (19, 20). The programmable nuclease Cas9 was subsequently characterized and rapidly adapted for genome engineering across organisms (21–23), followed by Cas12a as a complementary single-effector nuclease with distinct PAM and cleavage properties (24, 25). Although much engineering has relied on Class 2 effectors, such as Cas9 and Cas12, Type I systems are the most prevalent CRISPR-Cas systems in nature by a substantial margin (26, 27), suggesting an opportunity to repurpose endogenous effectors for editing, particularly in strains that are difficult to transform. Type I systems, the most abundant type in nature, are characterized by a multiprotein effector complex known as Cascade, which binds a crRNA to direct target recognition and recruits the exonuclease-helicase Cas3, leading to unwinding and processive degradation of the target DNA (28–33). In contrast, Class 2 systems, including Type II and Type V, employ a single multidomain effector protein—the signature endonucleases Cas9 and Cas12, respectively (31–33)—guided by a crRNA-tracrRNA duplex, to introduce defined double-strand breaks with blunt ends for Cas9 (20, 21, 34) and staggered ends for Cas12 at specific target sites (24, 35). Within lactic acid bacteria, endogenous CRISPR-Cas systems have been repurposed for practical applications (36), including CRISPR repeat- and spacer-based strain typing in *Bifidobacterium* (37), Cas9-mediated self-targeting in *Streptococcus thermophilus* to select excision of large genomic islands (38), and self-targeting in *Lactobacillus gasseri* using sgRNA mutagenesis to probe guide requirements (39). The first demonstration that endogenous CRISPR-Cas can be used for genome editing in a recalcitrant probiotic species was in *Lactobacillus crispatus*, using its native Type I-E system (40). A similar approach was later applied in *B. lactis* DSM10140, repurposing the endogenous Type I-G system to achieve genome editing, providing a foundation for our optimization across recalcitrant commercial *B. lactis* strains (41).

In this study, we develop and validate a widely applicable genome editing framework for *Bifidobacterium animalis* subsp. *lactis* that leverages its native Type I-G CRISPR-Cas system. We redesigned the editing plasmid backbone to improve delivery across commercial strains, tested different spacers to account for variable targeting activity, and implemented short and long homology arms to support recombination, demonstrating feasibility by generating knockouts at three loci. This platform is intended to enable routine genetics in *B. lactis*, to establish the genetic basis for probiotic phenotypes, and to open a path toward engineering live biotherapeutic strains with enhanced attributes.

## RESULTS

### Prevalence of CRISPR-Cas systems across *Bifidobacterium lactis* genomes

**Fig 1:**
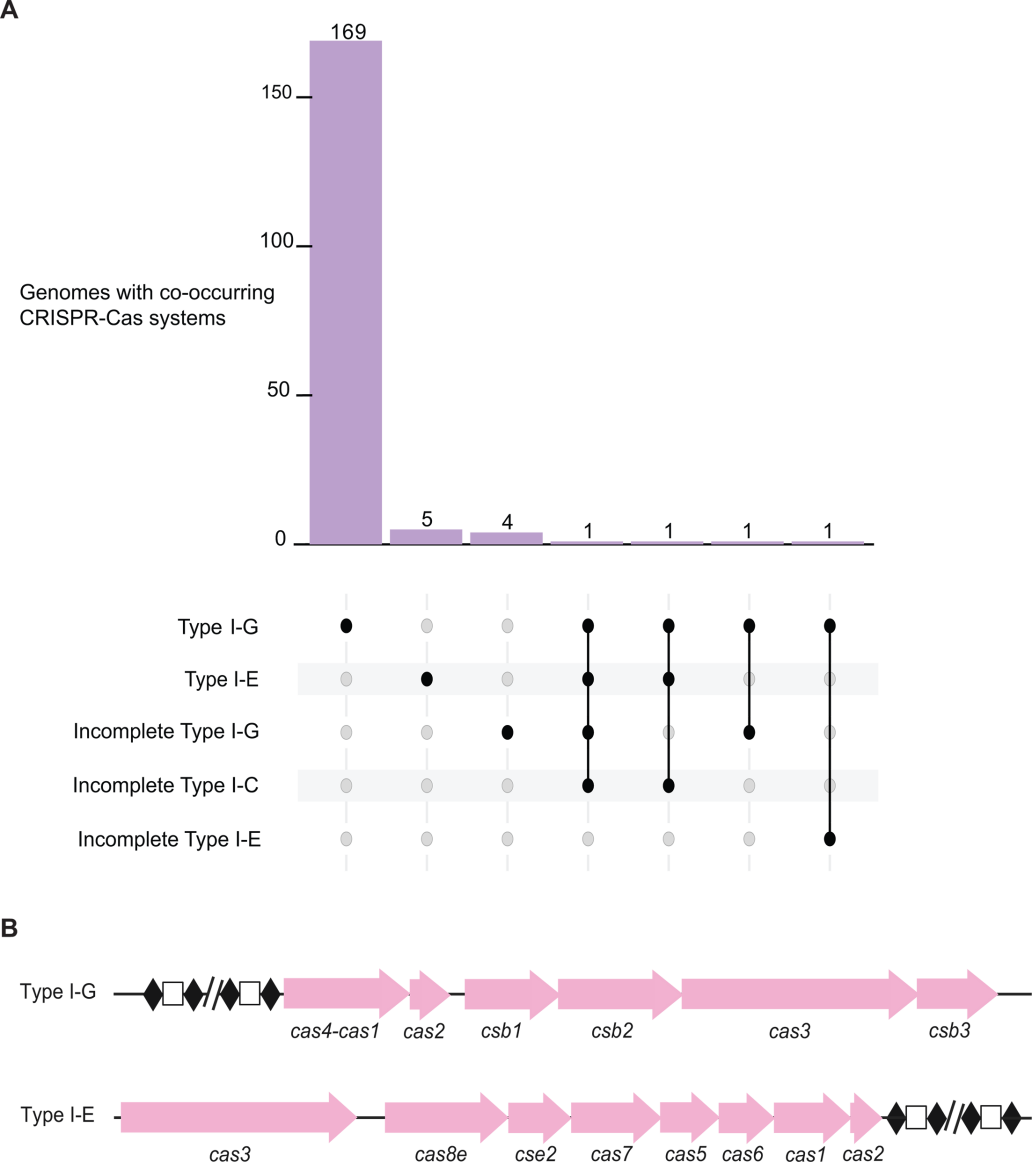
Distribution and locus architecture of CRISPR-Cas systems in *Bifidobacterium animalis* subsp. *lactis*. (**A**) A system prevalence and co-occurrence across genomes. UpSet plot summarizing the distribution of CRISPR-Cas subtypes detected in *B. lactis*. The intersection bars and connected dots indicate genomes harboring multiple systems and their specific combinations. (**B**) Architecture of the predominant endogenous systems. Schematic locus organization of Type I-G and Type I-E systems.

We analyzed 182 publicly available *B. animalis* subsp. *lactis* genomes using CRISPRCasTyper (42), a software tool that detects *cas* genes and CRISPR arrays and predicts the corresponding CRISPR-Cas system types and subtypes, to determine CRISPR-Cas occurrence and classification. We found that complete Type I-G systems predominated (~95%) (31–33, 43), whereas complete Type I-E systems were relatively rare (~4%) (Fig. 1A; Table S1) (28, 31–33), with overall conserved architecture (Fig. 1B). According to the criteria established by Makarova et al. in the latest classification study, the Type I-E CRISPR-Cas loci in our data set most closely match the canonical I-E1 subtype (33). However, we also detected a handful of incomplete Type I-G systems, defined by the absence of one or more *cas* genes or the CRISPR array (Fig. 1A), reflecting either genuinely degenerate systems or sequencing and assembly artifacts. Additionally, incomplete Type I-C (31–33, 44) or Type I-E systems (28, 31–33) occasionally co-occurred with complete CRISPR-Cas systems within the same genome (Fig. 1A). Among the seven genomes with complete Type I-E systems, four possessed a single *cas* operon and multiple adjacent CRISPR arrays, suggesting a single functional system with multiple arrays. Notably, two genomes carried both complete Type I-G and Type I-E systems along with other incomplete loci (Fig. 1A). Together, these results indicate that Type I-G is the canonical system in *B. lactis*, whereas Type I-E occurs in a small subset, and mosaic or incomplete loci appear sporadically.

### Conservation of Cas1 and Cas3 in *Bifidobacterium lactis*

**Fig 2:**
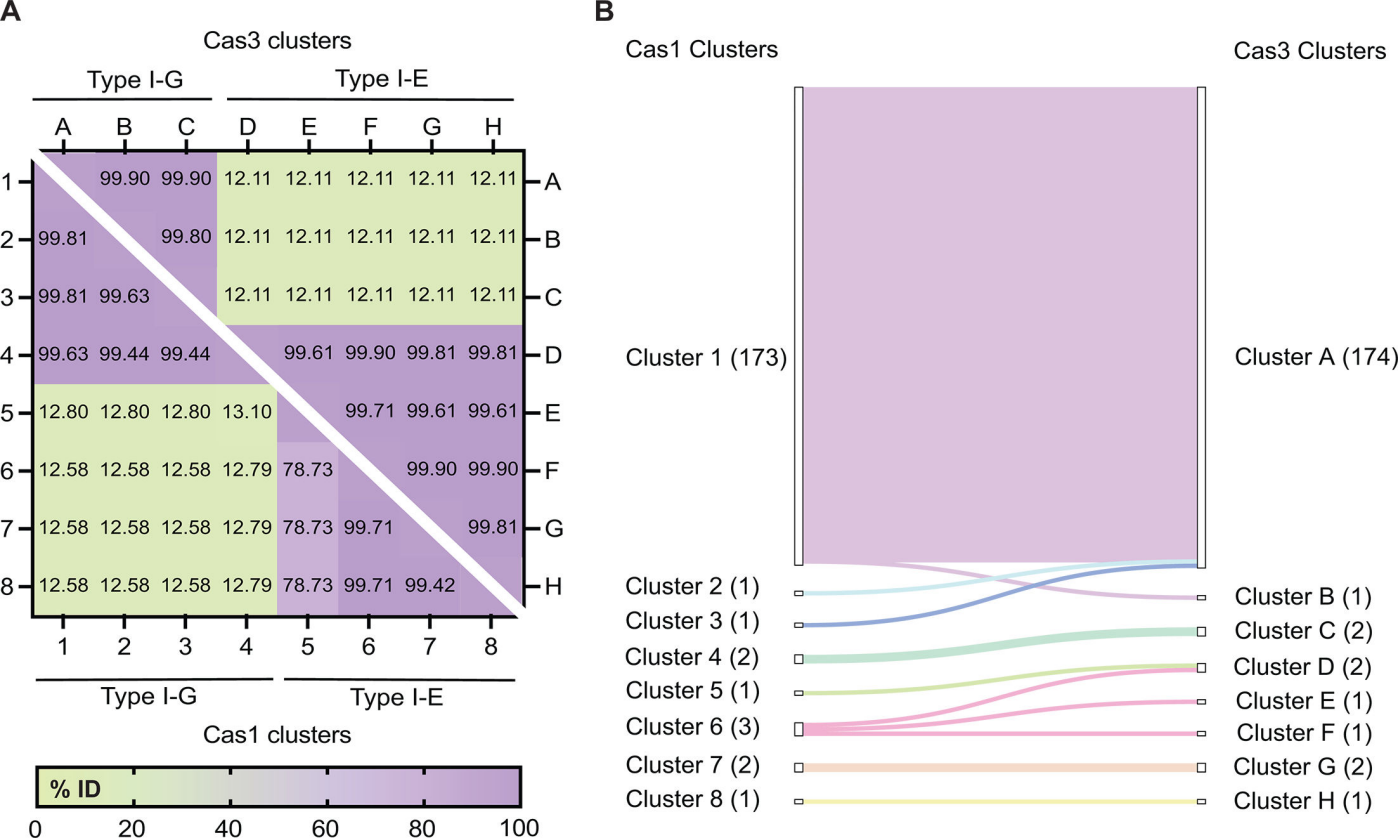
Identity-based Cas1 and Cas3 protein clusters and their correspondence. (**A**) Pairwise identity matrices for Cas1 and Cas3 proteins. Heatmaps show amino acid identity for nonredundant protein sets after collapsing sequences that are 100% identical. The association of the clusters with the CRISPR-Cas subtypes (Type I-G or Type I-E) is also indicated. (**B**) Cas1-Cas3 protein-cluster concordance. Sankey diagram mapping genome membership from Cas1 to Cas3 protein clusters. Node labels report cluster ID and size (n), and ribbon widths are proportional to genome counts. The flows highlight agreement and reassortment between the amino acid-based clustering analyses.

To assess the diversity of CRISPR-Cas systems within *B. lactis*, which exclusively harbors Type I systems (specifically Type I-G and Type I-E), we generated identity-based distance matrices from the amino acid sequences of the ubiquitous Cas1 and Type I signature Cas3 proteins (Fig. 2A). Cas1, the conserved integrase for spacer acquisition and a universal marker of CRISPR-Cas systems, was selected for its evolutionary conservation and its importance in system classification (31). Cas3, the helicase-exonuclease essential for target degradation and the signature feature of Type I systems, was included in the analysis to assess variation in interference machinery (28–30). Notably, in Type I-G systems, a Cas1-Cas4 fusion is encoded, which differs substantially from canonical Cas1 in other subtypes (32, 33). All genomes contained at least one Cas1 and one Cas3, with the exception of GCA_028203055.1, which harbored an incomplete Type I-G system lacking Cas3 (Tables S1 and S2). In contrast, the two genomes with both complete Type I-G and I-E systems (GCA_004154645.1 and GCA_004154425.1) encoded distinct Cas1 and Cas3 proteins for each system (Tables S1 and S2).

Because many Cas1 and Cas3 proteins were identical in sequence, we first clustered them at 100% identity. This produced eight Cas1 clusters, four associated with Type I-G systems, and four with Type I-E (Fig. 2A; Table S2). Cas3 sequences also formed eight clusters, three linked to Type I-G and five to Type I-E systems (Fig. 2A; Table S2). Within each subtype, Cas1 and Cas3 proteins were highly conserved with greater than 99% identity, except for a divergent Type I-E cluster (Cluster 5) that showed ~79% identity to the other I-E clusters (Clusters 6–8) (Fig. 2A). Between subtypes, Cas1 shared only about 13% identity, and Cas3 about 12% identity (Fig. 2A).

To relate Cas1-based groupings to Cas3, we mapped genomes between the two clustering analyses with a Sankey diagram (Fig. 2B). Of 177 Cas1 proteins associated with Type I-G systems, 173 grouped within a single cluster (Cluster 1), indicating a high degree of conservation (Fig. 2B). Clusters 2 and 3 each contained one Cas1, and Cluster 4 contained two. Genomes in Cas1 Clusters 1, 2, and 3 encoded Cas3s in Cluster A, except for one genome with a highly similar Cas3 in Cluster B (Fig. 2B). Type I-G–associated Cas1 Cluster 4 and Cas3 Cluster C were comprised of the same two genomes that also carried complete Type I-E CRISPR-Cas systems (Fig. 2B). In those genomes, the Type I-E proteins grouped in Cas1 Cluster 7 and Cas3 Cluster G (Fig. 2B). Among the seven genomes with Type I-E systems, we observed four distinct Cas1 clusters and five Cas3 clusters (Fig. 2A and B). This pattern contrasts with the high conservation across Type I-G systems and indicates greater protein-level diversity among *B. lactis* strains with Type I-E systems.

### CRISPR array organization and spacer content in *B. lactis*

**Fig 3:**
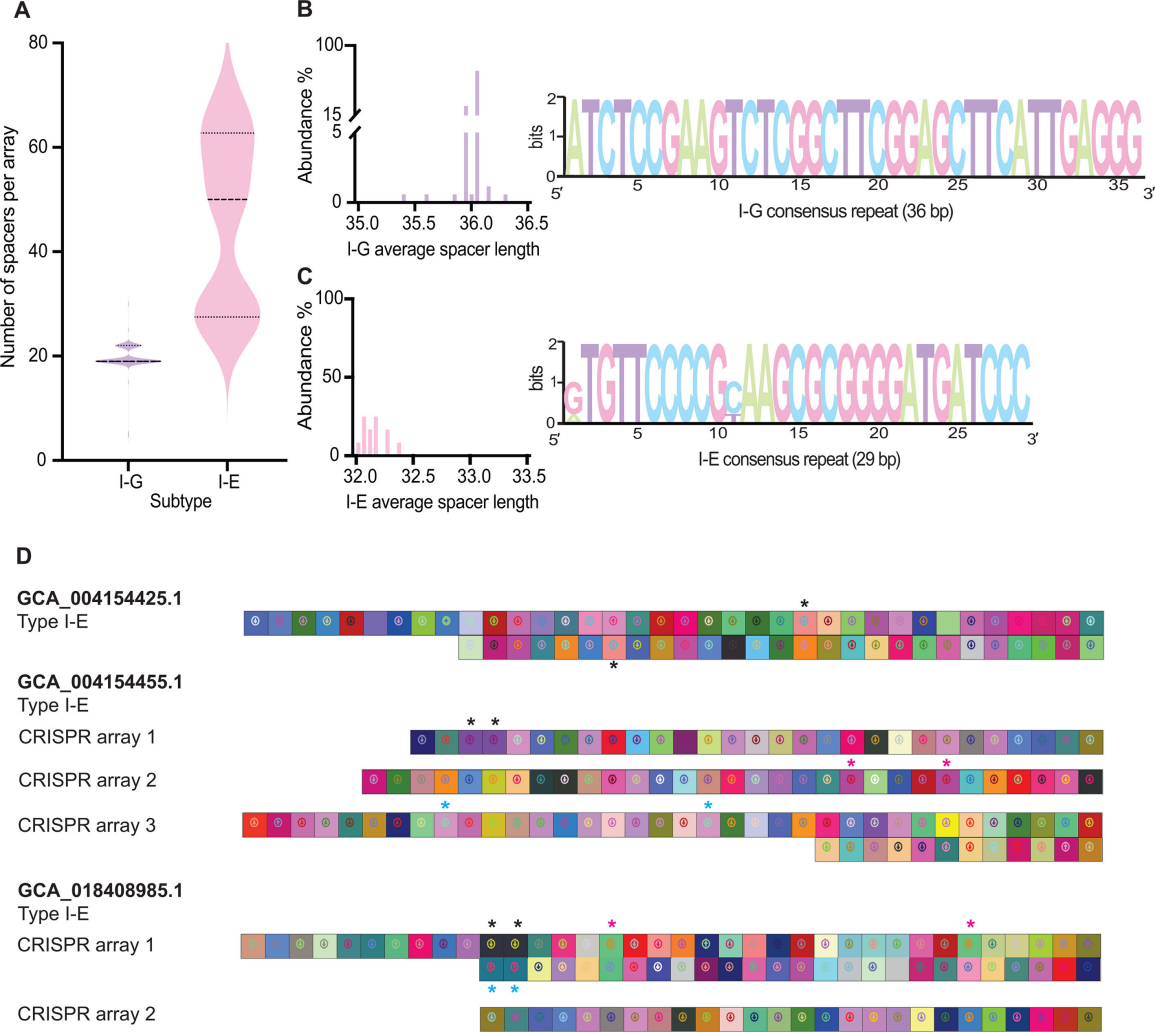
Spacer profiles and repeat motifs across CRISPR-Cas subtype. (**A**) Spacers per array by subtype. Distribution of the number of spacers per CRISPR array for Type I-G and Type I-E CRISPR-Cas systems. Dotted lines mark the first and third quartiles, and the dashed line marks the median. (**B**) Type I-G spacer length and repeat. Distribution of Type I-G spacer lengths and a WebLogo of the Type I-G consensus repeat sequence (36 bp). (**C**) Type I-E spacer length and repeat. Distribution of Type I-E spacer lengths and a WebLogo of the Type I-E consensus repeat sequence (29 bp). (**D**) Repeated spacers within arrays. CRISPRviz graphs highlighting representative arrays that contain repeated spacer sequences for selected CRISPR-Cas loci. Recurring spacers within the same genome are marked with asterisks of the same color.

We next examined CRISPR arrays from complete CRISPR-Cas systems in *B. lactis*, focusing on repeats and spacer content, the defining and adaptive feature of these immune systems. As a proxy for the CRISPR array length, the number of spacers per array varied more widely in Type I-E systems, with some arrays approaching 70 spacers—substantially longer than the typical CRISPR array size, indicative of their adaptive immunity potential and activity—whereas Type I-G systems were more uniform in size, most often at 19 or 22 spacers (Fig. 3A) (45). Spacer length distributions also differed between subtypes. Type I-G arrays clustered tightly, with most arrays having an average spacer length close to the prototypical 36 bp (Fig. 3B). Type I-E arrays had average spacer lengths close to 32 bp, which is longer than the common 29 nt length (Fig. 3C) (46).

Beyond spacer number and length, analysis of spacer identity and organization revealed striking patterns of conservation within *B. lactis* Type I-G CRISPR arrays (Table S4). Despite analyzing hundreds of genomes, nearly all Type I-G arrays consisted of either 19 or 22 spacers, consistent with their uniform lengths described above. Remarkably, 116 genomes with 19 spacers carried an identical CRISPR array, sharing the same spacer repertoire and order (Fig. S1A and B). Even among genomes with 19-spacer arrays that differed from the dominant configuration, variation was limited to only one or two spacers, underscoring the high level of conservation. Genomes with 22-spacer arrays also showed strong consistency, with all but one sharing the same array structure, which corresponded to the conserved 19-spacer array with three additional spacers acquired in the middle of the array (Fig. S1A and B). SpacerPlacer reconstructions further highlighted this stability, revealing only a small number of putative spacer acquisitions, deletions, or duplications across the Type I-G arrays analyzed (Fig. S1A). In contrast, Type I-E arrays displayed extensive spacer diversity, with little conservation of spacer identity or order across genomes. No spacers were shared between Type I-G and Type I-E CRISPR-Cas systems, indicating fully distinct spacer pools and independent adaptive histories for the two subtypes.

We then evaluated repeat features. Repeat length and consensus repeat sequence were highly conserved within each subtype. Type I-G repeats were 36 bp across all genomes, with an identical consensus sequence observed in every instance (Fig. 3B). Type I-E repeats were 29 bp long, with only two variable positions in the consensus sequence, namely G or A at position 1 and C or T at position 11 (Fig. 3C).

To investigate potential self-targeting, we queried every spacer against its source genome. We did not detect any self-targeting event, again reflecting the adaptive nature of these immune systems targeting invasive genetic elements. Instead, we identified recurring spacer sequences in several arrays, predominantly in Type I-E systems and in one Type I-G system (Table S3). At least six genomes contained repeated spacers, some identical and others differing by only a few nucleotides (Table S3). To determine whether near-identical spacers could target the same protospacer, we considered both sequence identity and the position of mismatches. We defined the seed region as the 5′ segment adjacent to the PAM within the protospacer, including positions 1 through 8, and used differences within this region to classify spacers as functionally distinct (47). Differences outside this region were considered unlikely to affect targeting, and such spacers were treated as functionally equivalent. As shown in Fig. 3D and Table S3, some systems contained a single repeated spacer, while others carried multiple instances within the same array. In Type I-E systems with one *cas* operon but multiple CRISPR arrays, repeated spacers were sometimes shared across all arrays and, in other cases, limited to a single array. Repeated spacers appeared either adjacent positions or dispersed at distant positions within the array.

### Endogenous type I-G CRISPR-Cas system and editing vector design

**Fig 4:**
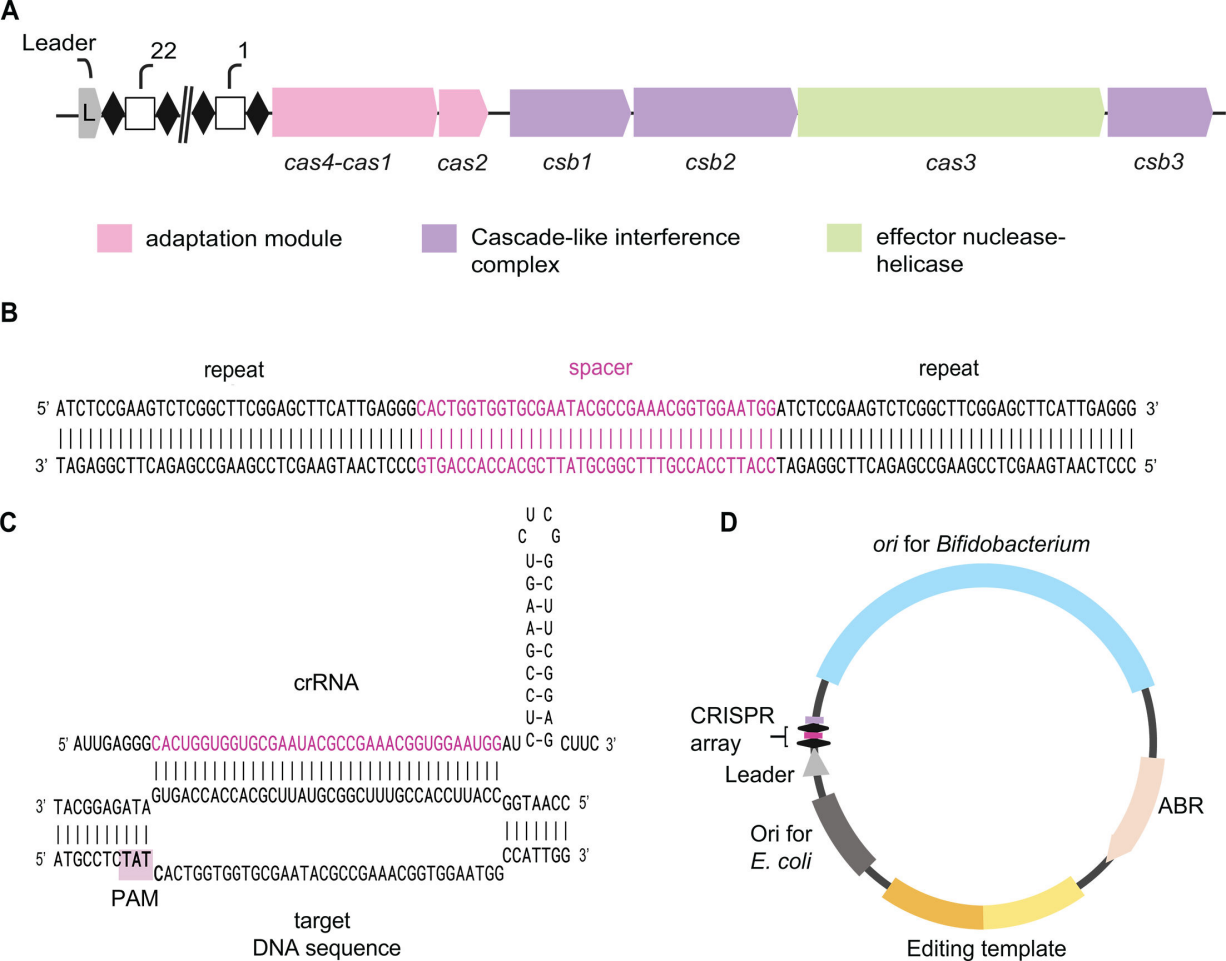
Endogenous Type I-G CRISPR-Cas locus in *B. lactis* Bl-04 and CRISPR-editing vector design. (**A**) Locus architecture. Leader sequence, CRISPR array with spacer count, and the *cas* operon are shown. Genes are grouped by function: adaptation (*cas4-cas1* fusion and *cas2*), interference complex (*csb1*, *csb2*, *csb3*), and the *cas3* nuclease-helicase. (**B**) Repeat-spacer-repeat unit. Representative array segment with 5′−3′ orientation. (**C**) crRNA-target pairing and protospacer adjacent motif (PAM). Predicted crRNA secondary structure and base pairing of the spacer to the protospacer, with the adjacent PAM indicated. (**D**) CRISPR-editing vector design. Plasmid map with origins of replication (*ori*) for *E. coli* and *Bifidobacterium*, an antibiotic resistance marker (ABR) for selection, a synthetic CRISPR array driven by the leader, and a homologous editing template.

Most *B. lactis* strains (>95%) encode a Type I-G CRISPR-Cas system. We thus leveraged this conserved native machinery to generate targeted knockouts in the probiotic strain *B. lactis* Bl-04, which is widely used commercially, tolerates various environmental stresses and ingestion, and has reported benefits in human studies (48). The Bl-04 Type I-G CRISPR locus comprises an adaptation module with a *cas4-cas1* fusion and a *cas2*, and an interference complex (Cascade; Csb1, Csb2, Csb3) that recognizes the 5′-TAT-3′ PAM and binds DNA targets via crRNA-DNA base pairing (Fig. 4A through C) (41). Cas3, a helicase-exonuclease, is recruited to degrade the target DNA. Using this functionally characterized system (41), we avoid the need to express heterologous Cas proteins, which is advantageous for strains that are otherwise recalcitrant to transformation, streamlines the editing construct process, and relies on host cell machinery.

To redirect the native machinery, we designed a synthetic CRISPR array that mirrored the architecture of the native array, encompassing the native leader promoter sequence, two consensus CRISPR repeats flanking a targeting spacer of the modal native spacer length, and a transcriptional terminator preventing read through by RNA polymerase (Fig. 4D). To enable allelic replacement, we concurrently supplied an editing template composed of homology arms flanking the intended knockout region. In this design, homologous recombination produces the edited allele, and any wild-type alleles are then targeted by the crRNA-Cascade complex, with Cas3 degrading the DNA for counterselection of non-edited wild-type genotypes. The editing plasmid also contained an origin of replication for *E. coli* for cloning and propagation, an origin of replication functional in *B. lactis*, and an antibiotic resistance marker for selection in both hosts (Fig. 4D).

### Optimization of CRISPR vector backbone for improved transformation efficiency

**Fig 5:**
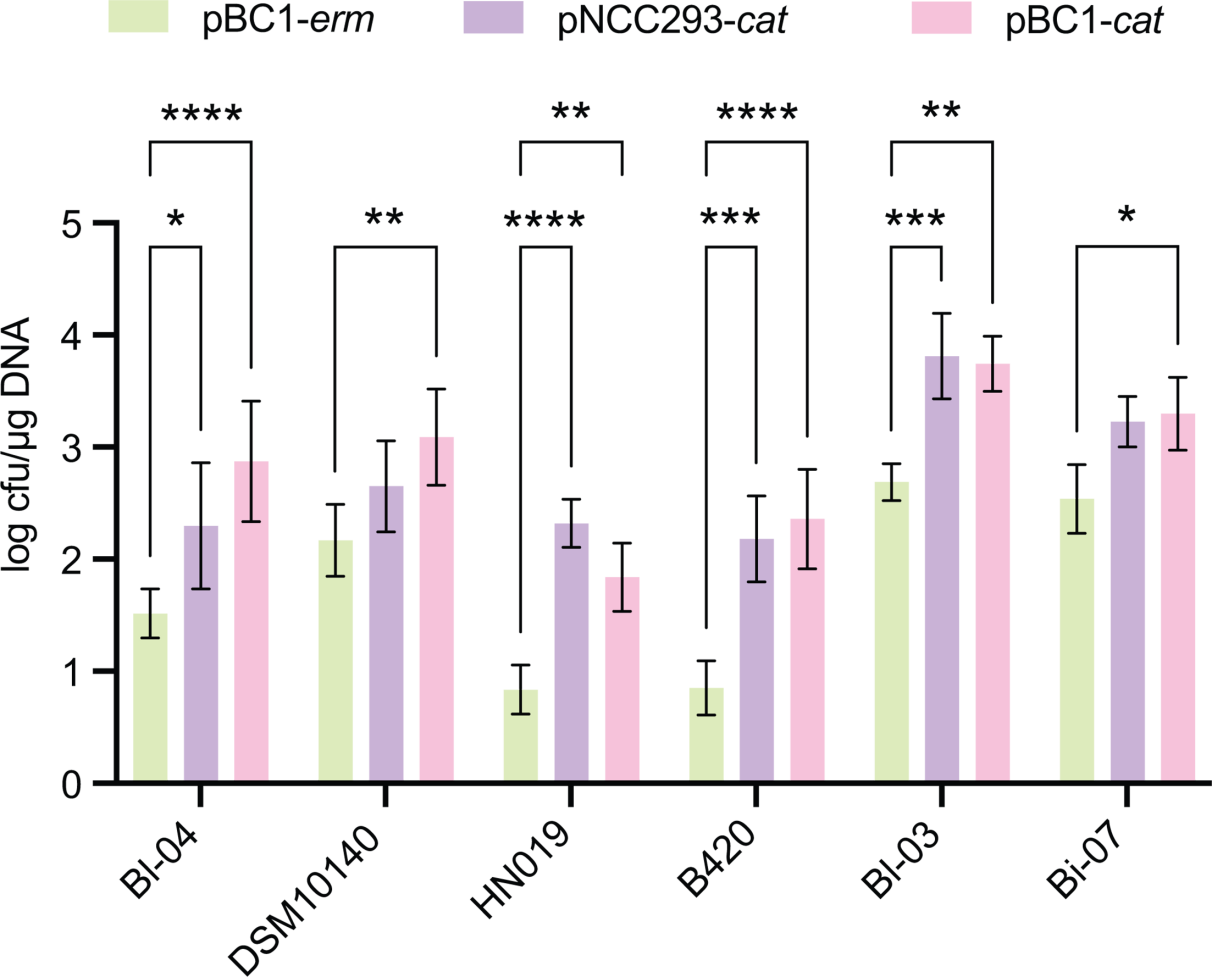
Backbone vector optimization across six commercial *B. lactis* strains using three designs with different replicons and antibiotic resistance markers. Transformation efficiencies are reported as log_10_ cfu/μg DNA for three backbone designs with different *Bifidobacterium* origins of replication and antibiotic resistance markers. Each bar shows mean ± SD of *n* = 3 independent experiments. Statistical testing was performed using two-way ANOVA. Significant differences are denoted as * *P* < 0.05, ** *P* < 0.01, *** *P* < 0.001, and **** *P* < 0.0001.

Transformation of *B. lactis* is challenging because of the multiple restriction-modification (R-M) systems and the relatively thick bifidobacterial cell wall (11). Plasmid features, such as the origin of replication (*ori*) and antibiotic resistance markers, can strongly influence transformation efficiency (49). To identify a more broadly functional backbone for our CRISPR-editing vector, we compared three plasmids with different *ori*-marker combinations: the previously reported pBC1-*erm* (pTRK1278) plasmid (41), a variant carrying pBC1 with a chloramphenicol resistance marker (pBC1-*cat*, pTRK1400), and a plasmid with the *B. longum*-derived pNCC293 *ori* and chloramphenicol resistance (pNCC293-*cat*, pTRK1401). We determined transformation across six commercial *B. lactis* strains (Fig. 5). We observed consistently low transformation efficiency with the original pBC1-*erm* plasmid in all strains (Fig. 5). In contrast, vectors with chloramphenicol resistance showed markedly higher efficiencies (Fig. 5). In *B. lactis* Bl-04, the strain selected for genetic engineering, pBC1-*cat* improved transformation by more than 20-fold relative to pBC1-*erm* (Fig. 5). The pNCC293-*cat* plasmid also increased transformation efficiency in most strains, besides DSM10140 and Bi-07, and it outperformed pBC1-*cat* in a couple of strains (Fig. 5). Since pBC1-*cat* consistently enabled significantly higher transformation efficiency than pBC1-*erm* across all strains (Fig. 5), we selected pBC1-*cat* as the backbone for subsequent construction of our CRISPR-editing vectors.

### Design of gene deletion constructs and target site mapping

**Fig 6:**
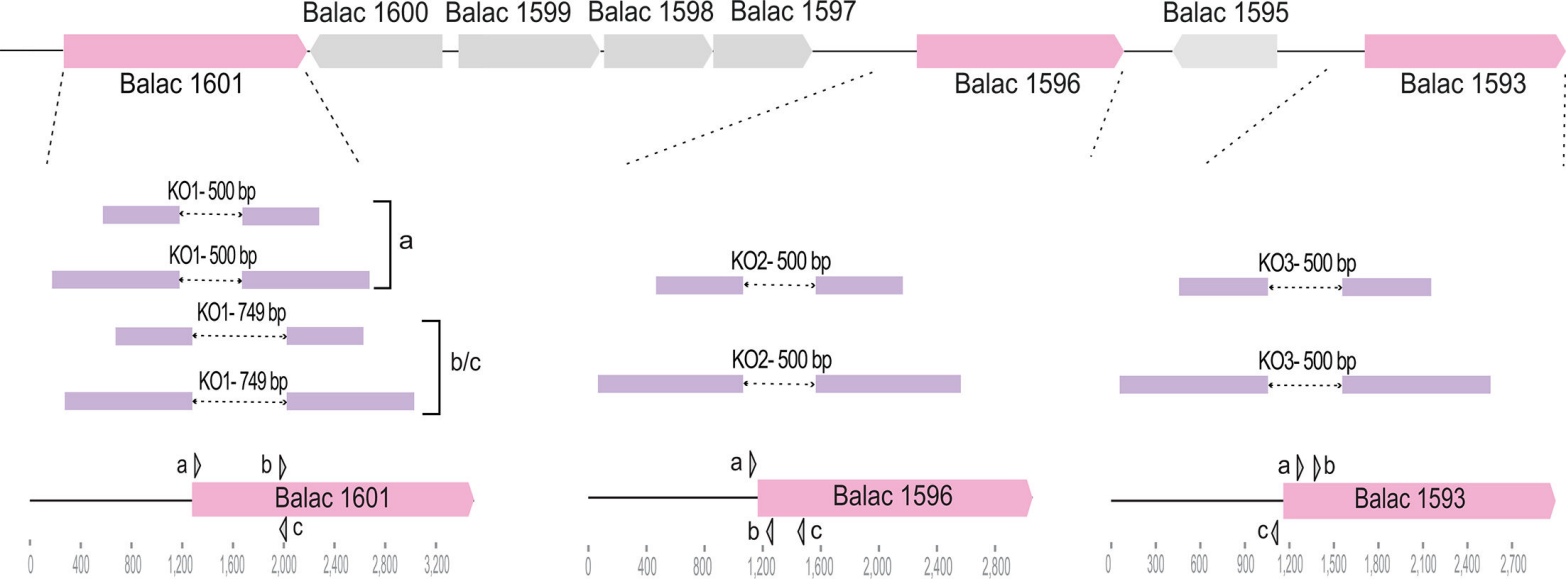
Editing template design and protospacer/target mapping. Locus map in *B. animalis* subsp. *lactis* Bl-04 with targeted glycoside hydrolases highlighted (pink). Target sites are expanded as zoomed schematics, showing intended deletion lengths (dotted arrows), protospacer positions corresponding to spacers a-c used in the CRISPR-editing vectors (gray arrows), and homology arm lengths in the editing templates (purple bars). Two template sizes were tested: short with 600 bp flanking arms (1.2 kb total), and long with 1,000 bp flanking arms (2 kb total). For the Balac 1601 target, designs using spacer a are compared with designs using spacers b and c.

We selected three glycoside hydrolases within a gene cluster implicated in the utilization of raffinose-family oligosaccharides (RFOs) as well as isomaltose, melibiose, and panose, and we used them as proof-of-concept targets to test the broad applicability of our editing strategy (Fig. 6). KO1 targeted Balac 1601, annotated as a GH36 α-galactosidase predicted to hydrolyze α−1,6-galactosidic bonds (Fig. 6) (50). KO2 targeted Balac 1596, hypothesized to encode a GH36 α-galactosidase (Fig. 6) (50). KO3 targeted Balac 1593, annotated as a GH13 α-glucosidase associated with α−1,6-glucosidic bond hydrolysis (Fig. 6) (50).

For each target gene, we designed three distinct spacers, and we paired each spacer with two editing templates to compare homology arm length recombination efficiency. The short and long templates used 600 bp and 1,000 bp homology arms, respectively (Fig. 6). We planned 500 bp deletions that spanned ~100 bp upstream of the start codon to disrupt promoter and proximal regulatory sequences and ~400 bp within the coding region to abrogate transcription (Fig. 6). KO1 was an exception, because PAM availability placed two protospacers further inside the ORF, yielding a 749 bp deletion (Fig. 6).

Spacer positions were dictated by PAM availability in the target region. For each spacer, the 35 nt sequence immediately downstream of the PAM constituted the protospacer, which is complementary to the spacer encoded in the synthetic CRISPR array. By design, we included spacers with matching protospacers residing on either the coding or non-coding strand to test strand dependency in this system (Fig. 6).

For each intended knockout, we transformed six CRISPR-editing plasmids, which correspond to three spacers in combination with two editing template lengths. In parallel, we transformed CRISPR-screening plasmids carrying the same artificial arrays but lacking an editing template in order to select for potential naturally emerging mutants. The goal of this design was to assess feasibility across loci and to evaluate template length requirements rather than to provide a quantitative comparison of targeting efficiencies.

### Validation of genome edits across multiple loci

**Fig 7:**
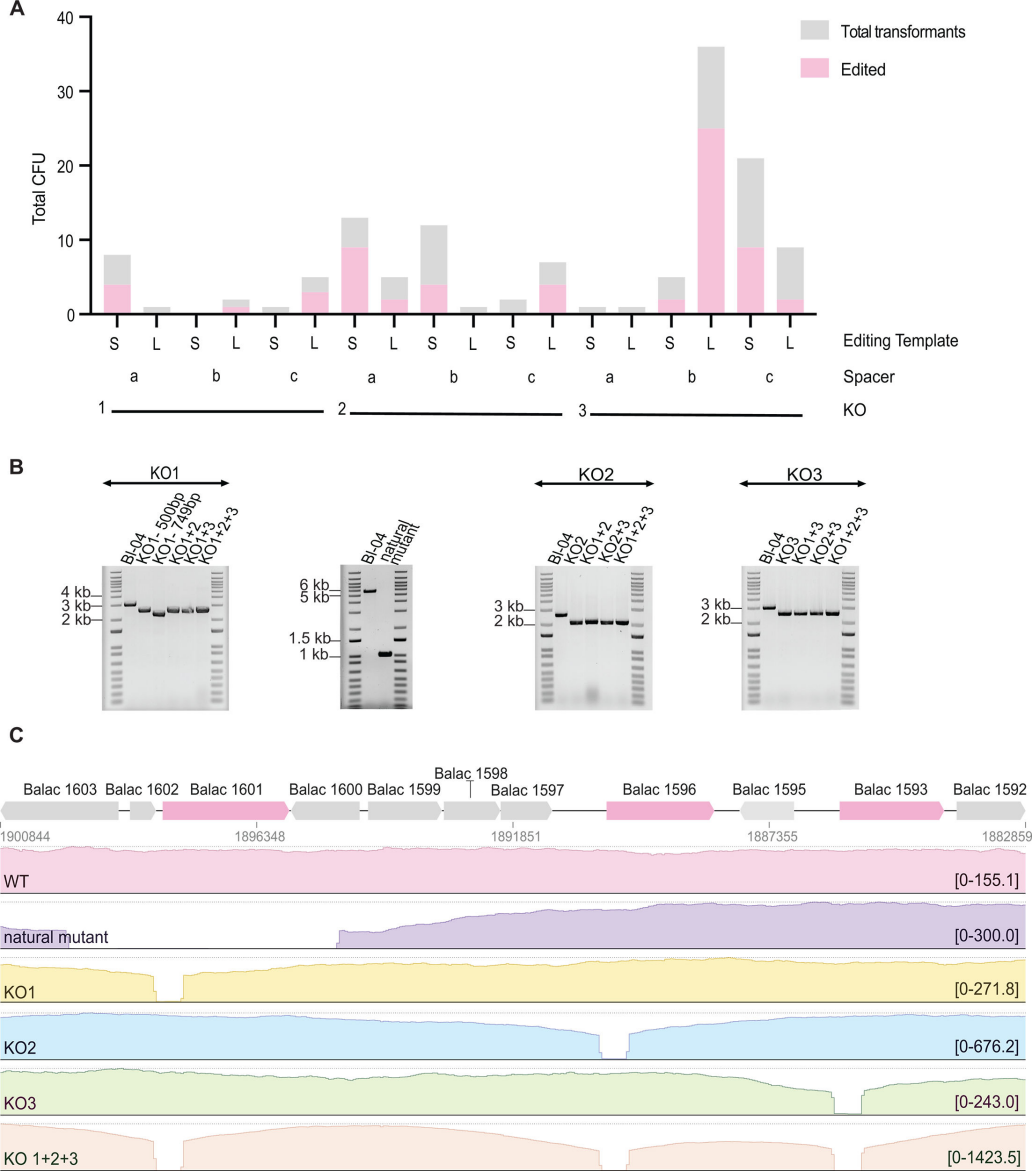
Editing outcomes and validation of knockouts in *B. lactis* Bl-04. (**A**) Transformant yield and edited fraction per CRISPR-editing design. Stacked bars show total transformants (gray) and the edited subset (pink) for KO1, KO2, and KO3 using spacers a–c and two editing template lengths (short, 1.2 kb; long, 2.0 kb). Results reflect a single experimental trial. (**B**) PCR screening of edited isolates. Agarose gels for single knockouts (KO1, KO2, KO3) and sequential combinations (KO1+2, KO1+3, KO2+3, KO1+2 + 3). A naturally occurring mutant identified during screening with spacer a for Balac 1601 is included. (**C**) Oxford Nanopore (ONT) whole-genome sequencing of the edited isolates. Coverage plots for wild-type and edited isolates highlighting the deletions at the targeted loci.

We quantified total transformants and the edited subset for KO1, KO2, and KO3, using CRISPR-editing vectors with the corresponding spacers (a, b, c), and the two editing template lengths (short, 1.2 kb; long, 2.0 kb) (Fig. 7A). The results represent a single experimental trial and should therefore be interpreted accordingly. Nevertheless, we observed clear patterns that may inform future editing efforts. In particular, testing more than one spacer and more than one editing template length increased the likelihood of success. For example, no edited transformants were obtained for KO3 when using spacer a, whereas numerous edits were achieved with spacers b and c (Fig. 7A), reflecting potential variable spacer efficiency. Additionally, spacers whose protospacers resided on either the coding or non-coding strand both yielded successful edits, indicating that the target strand did not preclude editing under the conditions tested. In most cases, the short editing template was sufficient to mediate recombination, but longer templates were occasionally preferred or even required. For instance, the larger 749 bp deletion in KO1 (Balac 1601) was only recovered when the long editing template was used (Fig. 7A).

To verify edits, we screened colonies by PCR using primers that hybridize outside the homology arms to ensure that only correctly edited alleles were detected, rather than plasmid-borne sequences (Fig. 7B; Table S6). In addition to the designed knockouts, we also recovered a natural mutant when using the CRISPR-screening vector that carried spacer a for KO1 but no editing template. Across the transformants obtained with the CRISPR-based screening vectors lacking an editing template, summarized in Table S7, this was the only instance in which a natural mutant was identified. This mutant harbored a 4,743 bp deletion spanning part of Balac 1603, the entirety of Balac 1602 and Balac 1601, and part of Balac 1600 (Fig. 7B). Sequence analysis indicated that the deletion arose through microhomology-mediated excision at an 8 bp repeat, reflecting the potential to use CRISPR as a screening modality to select for rare naturally occurring mutants (38, 41).

After editing, we passaged the edited strains on non-selective medium to achieve plasmid curing. By the tenth passage, all screened colonies no longer carried the plasmid, confirming successful curing. This enabled sequential rounds of editing and demonstrated the feasibility of iteratively generating multiple knockouts in the same genetic background (Fig. 7B). Plasmid curing also removed the antibiotic resistance marker, which is advantageous for downstream applications. Finally, whole-genome sequencing of selected isolates confirmed that the edits were specific and predictable, with no detectable off-targets (Fig. 7C).

### Functional characterization of knockout mutants

**Fig 8:**
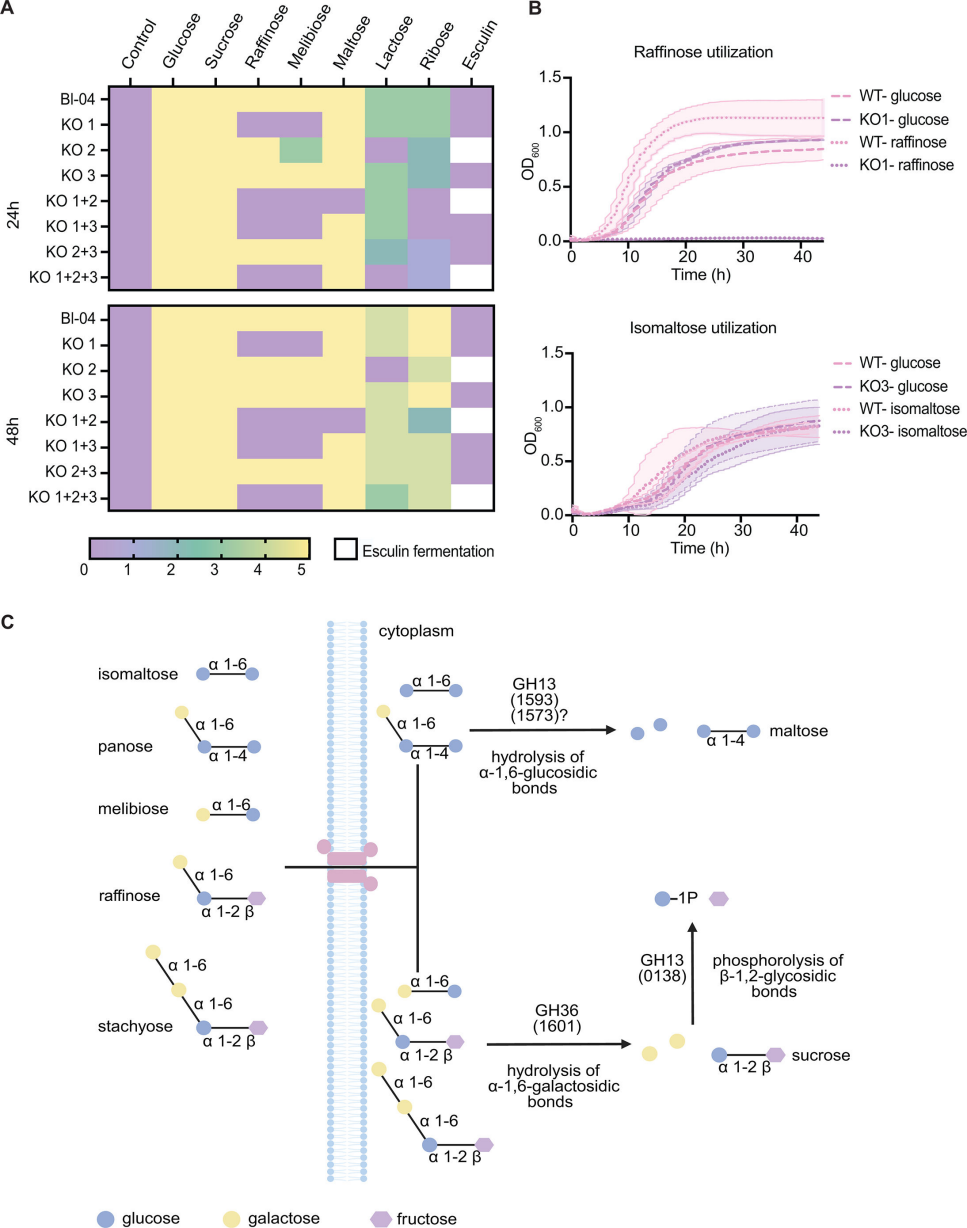
Carbohydrate utilization phenotypes for the edited isolates and pathway model. (**A**) API 50 CHL fermentation profiles. Heatmap for wild-type and edited isolates at 24 h and 48 h, showing only substrates with altered phenotypes. Color scale: purple indicates no fermentation, yellow indicates full fermentation, and intermediate shades indicate partial fermentation. (**B**) Growth on diagnostic substrates. Growth curves (OD₆₀₀ over time) for KO1 and wild-type in glucose and raffinose (top), and for KO3 and wild-type in glucose and isomaltose (bottom). Glucose serves as a growth control. (**C**) Proposed uptake of oligosaccharides and catabolic routes into the bifid shunt. Schematic of oligosaccharide transport and hydrolysis. The transporter is shown in pink. Enzymes are annotated by locus tag and glycoside hydrolase family (GH); linkage types are indicated.

To further validate the outcome of our CRISPR edits beyond PCR confirmation, we assessed the functional consequences of the deletions through phenotypic characterization. Since the targeted genes were annotated as sugar hydrolases, we compared carbohydrate utilization profiles of the edited strains (single and multiple knockouts) against the wild-type using the API 50 CHL assay (Fig. 8A). Disruption of Balac 1601 (KO1), annotated as a GH36 α-galactosidase predicted to hydrolyze α−1,6-galactosidic bonds, consistently abolished fermentation of raffinose and melibiose in all strains carrying this knockout (Fig. 8A and C). We then tested growth with raffinose as the sole carbon source and observed growth only for the wild-type, while KO1 mutants showed no growth, which confirmed the API result (Fig. 8B; Fig. S2). The knockout within Balac 1596 (KO2) produced a distinct phenotype, characterized by loss of lactose fermentation and gain of esculin utilization, which was unexpected given its annotation as a GH36 α-galactosidase (Fig. 8A). Deletion of Balac 1593 (KO3), annotated as a GH13 α-glucosidase that might act on α−1,6 linkages in isomaltose and panose, did not yield the expected phenotype (Fig. 8B and C). The API panel does not include isomaltose nor panose, and growth on isomaltose as the sole carbohydrate did not differ between KO3 mutants and the wild-type (Fig. 8A and B; Fig. S2). These observations suggest that Balac 1593 may be misannotated or that another genomically encoded α-glucosidase compensates for this loss.

Double and triple knockouts did not display strictly additive phenotypes, underscoring the complexity of carbohydrate metabolism. For example, KO1+2 lost raffinose and melibiose fermentation as expected and gained esculin utilization, but unlike KO2 alone, it retained the ability to ferment lactose and instead showed reduced fermentation of maltose and ribose (Fig. 8A). Similarly, KO2+3 displayed only reduced ribose fermentation without the lactose or esculin phenotypes seen in KO2 (Fig. 8A). These results highlight that disrupting single genes can have indirect and sometimes unpredictable effects on carbohydrate metabolism, and that combined knockouts may yield emergent phenotypes not inferred from single mutants alone.

## DISCUSSION

Genome editing in bifidobacteria has been hampered by low transformation efficiency, limited recombination machinery, and active restriction-modification systems (51). CRISPR-based approaches have nevertheless been demonstrated in *B. lactis*, *B. animalis*, *B. breve*, and *B. longum*, using both endogenous CRISPR-Cas systems and exogenous nucleases, such as Cas9 or Cas9-derived base editors (41, 52–54). The first report of CRISPR editing in *B. lactis* established that the endogenous Type I-G CRISPR-Cas system is functional and can mediate genome engineering (41). However, success was limited to the type strain DSM10140 and to a single precise knockout. Our study builds on this foundation by developing a platform that enables iterative CRISPR editing across multiple *B. lactis* strains, including commercial isolates, through optimization of the editing vectors.

Comparative genomics revealed that *B. animalis* subsp. *lactis* strains exhibit very low genomic variability, with ≥99.85% sequence identity across the subspecies (55). This conservation extends to their CRISPR-Cas systems. The vast majority of genomes analyzed contained a complete Type I-G CRISPR locus (37, 56), and the systems were highly uniform, with conserved Cas1 and Cas3 proteins, nearly identical repeats, and similar spacer lengths, as well as strong spacer-level conservation across genomes. Such uniformity strongly suggests that a single editing strategy could be broadly applicable across the subspecies, with only spacer and template customization needed for each target. We also noted occasional recurring spacers in some CRISPR arrays, a phenomenon previously attributed to duplication rather than independent acquisition (57–60). Even if some repeats arose from weak interference and multiple acquisition events, they were found mainly in Type I-E systems, which are not relevant to our Type I-G CRISPR-based strategy.

Type I-G CRISPR-Cas systems are simplified versions of the typical Type I systems (32, 61). They have fewer components and unique gene arrangements, such as a Cas1-Cas4 fusion likely involved in pre-spacer processing and PAM selection, dual RNases (Csb1 and Csb2) for crRNA maturation, and Csb3, which contributes to Cascade assembly without catalytic activity (32, 43, 61). Cas3 then complexes with Cascade to the DNA target and provides helicase-exonuclease function for target degradation (32, 41, 43). Our system recognizes a 5′-TAT-3′ PAM (41), consistent with the AT-rich motifs described for other Type I-G systems (43, 56, 62).

The reason we chose to exploit the endogenous system rather than introduce heterologous nucleases, such as Cas9 or Cas12a, is that large exogenous *cas* operons are difficult to deliver into bifidobacteria, which are already recalcitrant to transformation (11), and they can be toxic in cells, especially if they originate from different species or genera (63–65). Base editors are also relatively large and limited by their narrow editing window, as well as the requirement for suitable codons to create a premature stop codon within a coding frame, in which the availability of compatible PAMs may be limited (66). Although they have been tested in multiple *B. lactis* strains, successful editing has so far been achieved only in the type strain DSM10140 (41). By contrast, endogenous CRISPR-based editing vectors remain compact, consisting of an artificial CRISPR array and homologous arms, which makes them more suitable for broad delivery into *B. lactis*.

One of the primary obstacles to CRISPR editing in *B. lactis* is transformation efficiency. As the selection of the plasmid can affect transformability (49, 51), our work prioritized backbone optimization. While the previously used pTRK1278 backbone (41), which contains the pBC1 replicon (67) and an erythromycin resistance marker, performed relatively poorly across six commercial strains, alternative vectors carrying either the pNCC293 replicon (68) or a chloramphenicol resistance marker achieved markedly higher efficiencies. This pattern was not explained by the predicted restriction BanLI/BanLII sites, which were identical in pTRK1278 and pBC1-*cat* (one BanLI, six BanLII) but fewer in pNCC293-*cat* (one BanLI, two BanLII) (69), suggesting that replicon identity and antibiotic marker effects were the primary drivers. Practically, backbone optimization should precede R-M circumvention, with site removal (51), host methyltransferase disruption (54), or plasmid pre-methylation in *E. coli* (69, 70) or in a cell-free transcription and translation system (TXTL) (71), layered as needed.

Editing efficiency in our system ultimately depends on homologous recombination, which must occur before CRISPR-mediated counterselection screens against wild-type genotypes (1, 2). Prior studies reported successful edits with homology arms of approximately 500 bp to 3 kb (72, 73). Consistent with a trade-off between recombination efficiency and vector size, we observed that 600 bp arms generally sufficed while preserving transformability, whereas 1,000 bp arms were necessary for larger or more complex edits, as exemplified by the 749 bp deletion in KO1. These observations are in line with work in *B. longum* and *B. animalis* showing that longer arms increase recombination efficiency (52, 72). Because our data derive from a single experimental trial, we cannot draw strong conclusions about differences between arm lengths. Instances in which shorter arms performed as well as, or better than, longer ones likely reflect the reduced plasmid size and improved transformation efficiency of the corresponding vectors, rather than a *bona fide* preference for shorter homology arms. A more systematic evaluation of arm length would require separating recombination potential from CRISPR counterselection. This could be achieved by transforming plasmids that contain identical homology arms but carry a non-targeting spacer. Recombination can be further enhanced by supplying auxiliary functions, including λ-Red in *E. coli* (74), a P1 Red-like operon in *L. plantarum* WCFS1 (75), or RecT for ssDNA donor templates in lactic acid bacteria (76–78).

A second constraint is spacer interference strength. Spacers with excessive targeting activity can eliminate transformants before recombination occurs, whereas low activity spacers may permit survival of unedited clones. Although our editing experiments were conducted in a single trial, the trend we observed across spacers, including some very few surviving transformants and occasionally no edits, is consistent with excessive counterselection. Motivated by prior reports of strand-dependent outcomes in Cas9-mediated editing, where targeting the non-template strand can hinder repair (79), we included protospacers on both strands. Editing was achieved when targeting either strand, suggesting that in *B. lactis*—and potentially in other bacteria with a Type I-G system—protospacer selection can be guided primarily by PAM availability rather than target strand preference. Guide RNA design considerations also include the influence of guide length. While tuning guide length can modulate activity in Cas9 and Cas12 systems (80–83), spacer length in Type I-G CRISPR-Cas systems is linked to crRNA maturation and Cascade assembly, as the full spacer is retained during processing (61, 84). As a result, altering spacer length is not as readily adjustable in this system, although future exploration of architecture-compatible variations may be worthwhile. Introducing temporal control using inducible promoters or riboswitch-controlled activation, for example, SIBR-Cas, could delay interference and further improve editing efficiency (85–87).

In addition to enabling precise editing, our CRISPR strategy can also be used for screening. When no homologous donor template is supplied, CRISPR targeting acts as a counterselection that selects for naturally occurring variants and identifies strains lacking specific genomic islands, prophages, or plasmids (38, 41, 88). This could be deployed in settings in which regulatory frameworks restrict the use of genetically engineered organisms.

This study was designed as a proof of concept, so we prioritized targets with predicted, straightforward phenotypic readouts of carbohydrate utilization. Accordingly, we selected glycoside hydrolases within the Balac 1593 to 1601 cluster whose loss could be confirmed by simple carbohydrate utilization assays and growth curves. Balac 1601 is annotated as a GH36 α-galactosidase that hydrolyzes α−1,6 galactosidic bonds in raffinose family oligosaccharides (RFOs) (Fig. 8C) (50). Consistent with this role, Balac 1601 knockouts exhibited loss of raffinose and melibiose fermentation across edited strains, as measured by API assays, and failed to grow when raffinose served as the sole carbon source in growth-curve comparisons with the wild-type. Balac 1596 is annotated as a GH36 α-galactosidase and is encoded downstream of a predicted ABC transporter (50). Its deletion produced a coordinated shift in carbon use, rather than a specific loss of RFO metabolism, which is consistent with a role in carbon-source prioritization rather than a purely catalytic function. Prior data showing weak induction by RFOs support this interpretation (44). This remains a hypothesis and should be tested by complementation, biochemical characterization, and transcriptomic profiling across relevant carbohydrates. The third target, Balac 1593, encodes a GH13 α-glucosidase predicted to act on α−1,6 linkages (Fig. 8C) (50). Deleting Balac 1593 did not impair isomaltose utilization, which suggests enzymatic redundancy. During growth on isomaltose, transcript data from *B. lactis* Bl-04 show increased expression of Balac 1573 (50). Although Balac 1573 is annotated as a GH13 α-glucosidase with a preference for α−1,4 bonds, its closest BLASTP match is an α−1,6 glucosidase from *B. animalis* (WP_022543166.1), indicating a broader substrate scope that may include α−1,6 linkages. Redundant α-glucosidases have been reported in bifidobacteria (89, 90). Consistent with this framework, tBLASTn using α-glucosidases from *B. breve* and *B. adolescentis* mapped mainly to Balac 1593, whereas the broader-specificity AglB from *B. adolescentis* mapped to Balac 1573 (89, 90). Together, these observations support a model in which Balac 1573 compensates for the loss of Balac 1593 during growth on isomaltose (Fig. 8C). This interpretation should be tested by complementing Balac 1593, defining the substrate ranges of Balac 1573 and Balac 1593 through biochemical assays, performing growth tests on defined α glucosides, and constructing a Balac 1573 single knockout as well as a Balac 1593 and Balac 1573 double knockout.

Collectively, our results establish a generalizable strategy for genome editing in recalcitrant *B. lactis*. The conservation of Type I-G CRISPR-Cas systems across *B. lactis* genomes suggests that strains with an intact and functional locus are likely to be amenable to the same editing framework. We demonstrate endogenous Type I-G CRISPR editing beyond DSM10140 and in commercial isolates, achieve the largest deletion reported using the endogenous Type I-G system in *B. lactis* (749 bp), generate knockouts at multiple targets, and show efficient vector curing that enables iterative editing. In developing this approach, we compared plasmid backbones, tested multiple spacers to address variable targeting activity, and evaluated short versus longer homology arms to support recombination. This toolkit enables rigorous dissection of *B. lactis* metabolism and other probiotic traits in strains of industrial relevance, providing a practical foundation for engineering next-generation probiotics.

## MATERIALS AND METHODS

### CRISPR-Cas system analyses and visualization

*Bifidobacterium animalis* subsp. *lactis* genomes were obtained from the data set curated by Lugli et al. (55) and supplemented with additional genomes released in the NCBI Genome database through January 2025. In total, 182 genomes were analyzed. CRISPR-Cas systems were predicted from the downloaded assemblies using CRISPRCasTyper v1.8.0 with flags—keep_tmp (Table S1) (42). An Upset plot was made using the upSetR package in RStudio to visualize the presence and co-occurrence of CRISPR-Cas subtypes across genomes (91). Cas1 and Cas3 amino acid sequences from *B. lactis* CRISPR-Cas systems were extracted from CRISPRCasTyper output, clustered with CD-HIT (v4.8.1.) using a 100% sequence identity cutoff (Table S2) (92), aligned with Clustal Omega (93), and visualized as percent identity distance matrices in Geneious Prime 2025.1.2 (https://www.geneious.com). A Sankey diagram was generated to depict correspondence between genome membership in Cas1 and Cas3 clusters. From the same outputs, we also extracted CRISPR array features, such as the number of spacers, repeat length, spacer length, and consensus repeat sequence, for each CRISPR-Cas subtype. CRISPR repeat sequences and spacers were manually inspected for orientation and length by visualizing the CRISPR arrays with Geneious Prime 2025.1.2 (https://www.geneious.com) and corrected as needed. The corrected CRISPRCasTyper results are provided in Table S1. Bar plots were generated to visualize the distribution of spacer and repeat lengths across CRISPR-Cas system subtypes based on abundance data. Sequence logos were created to represent the consensus repeat sequence for each CRISPR-Cas subtype. To assess potential self-targeting and detect repeated spacer blocks (Table S3), spacers from each CRISPR-Cas system were queried against their source genomes with BLASTn (94) via a modified Python script (95), and representative examples of repeated spacers were visualized with CRISPRviz (96). To further characterize the CRISPR array architecture, each spacer nucleotide sequence was assigned a unique identifier, such that spacers with identical sequences across genomes received the same spacer ID. Using these IDs, we cataloged spacer composition and order within each CRISPR array (Table S4). For Type I-G CRISPR arrays, which exhibited extensive spacer conservation and shared spacer blocks across genomes, we used SpacerPlacer to reconstruct and visualize putative spacer acquisitions, losses, and duplications (Fig. S1A) and to generate representative spacer-composition profiles (Fig. S1B) (60).

### Bacterial strains and growth conditions

The bacterial strains used in this study are listed in Table S5. Bifidobacterial strains were cultivated in De Man-Rogosa-Sharpe (MRS; Difco, Franklin Lakes, NJ, USA) broth supplemented with 0.05% (wt/vol) L-cysteine- HCl (Sigma-Aldrich, St. Louis, MO, USA) in broth, or on MRS agar containing 0.25% (wt/vol) L-cysteine-HCl (MRSC), at 37°C under anaerobic conditions (80% N2, 10% CO2, 10% H2). *Escherichia coli* NEB 10-beta (NEB, Ipswich, MA, USA; Cat# C3019H), used as the cloning host, was grown in Brain Heart Infusion (BHI; Difco, Franklin Lakes, NJ, USA) broth at 37°C with shaking at 250 rpm or on BHI agar at 37°C. Transformants carrying plasmids with the chloramphenicol resistance gene were selected using chloramphenicol (Cm; Fisher, Pittsburgh, PA, USA) at 15 μg/mL for *E. coli* and 5 μg/mL for *Bifidobacterium*. For erythromycin-resistant transformants, erythromycin (Erm; Fisher, Pittsburgh, PA, USA) was used at 150 μg/mL for *E. coli* and 2.5 μg/mL for *Bifidobacterium*.

### DNA manipulation and bacterial transformation

Bacterial chromosomal DNA was extracted with the DNeasy PowerLyzer Microbial Kit (Qiagen, Hilden, Germany; Cat# 12255-50), and *E. coli* plasmid DNA was isolated using the CompactPrep Plasmid Midi Kit (Qiagen, Hilden, Germany; Cat# 12843), according to the manufacturer’s instructions. Restriction digests were performed with NEB enzymes, and DNA fragments for plasmid assembly were prepared using the NEBuilder HiFi DNA Assembly Master Mix (NEB, Ipswich, MA, USA; Cat# E2621S). Oligonucleotides (single- and double-stranded) were ordered from Integrated DNA Technologies (IDT, Coralville, IA, USA), and gene fragments were synthesized by Genewiz (Azenta Life Sciences, South Plainfield, NJ, USA) (Table S6). PCR amplifications for cloning were carried out with Q5 Hot Start High-Fidelity 2X Master Mix (NEB, Ipswich, MA, USA; Cat# M0494L), whereas screening of transformants used OneTaq DNA Polymerase (NEB, Ipswich, MA, USA; Cat# M0509L). PCR products and digested plasmids were analyzed by electrophoresis on 1% agarose gels in TAE buffer and visualized with ethidium bromide. DNA was purified using Monarch Gel Extraction (NEB, Ipswich, MA, USA; Cat# T1020) or PCR and DNA Cleanup Kits (NEB, Ipswich, MA, USA; Cat# T1030). PCR products and plasmid constructs were confirmed by Sanger sequencing (Genewiz from Azenta Life Sciences, South Plainfield, NJ, USA) or Oxford Nanopore sequencing (Plasmidsaurus, South San Francisco, CA, USA). Plasmids were transformed into chemically competent NEB 10-beta cells (NEB, Ipswich, MA, USA; Cat# C3019H) using the manufacturer’s standard protocol. *Bifidobacterium* transformation was performed as previously described (41). Resulting *B. lactis* knockouts were verified by whole-genome sequencing using Oxford Nanopore Technologies (ONT; Plasmidsaurus, South San Francisco, CA, USA). Reads were aligned to the *B. lactis* Bl-04 reference genome with Minimap2 v2.30-r1287 (97), and BAM files were sorted and indexed with Samtools v1.22 (98). Reads were filtered using a MAPQ cutoff of 20 and percent identity cutoff of 90%. Coverage files were generated using Bedtools v2.31.1 to produce bedGraph files (99) and visualized with Lovis4u v0.1.5 (100).

### Construction of backbone vectors

The nucleotide sequence of plasmid pDP870 (GenBank accession no. DQ834380.1) (68) was synthesized by Genewiz (Azenta Life Sciences, South Plainfield, NJ, USA) and used to generate pNCC293-*cat* (pTRK1401). The synthesized plasmid was digested with BamHI (NEB, Ipswich, MA, USA; Cat# R3136S) and EcoRI (NEB, Ipswich, MA, USA; Cat# R3101S) to replace the spectinomycin resistance gene with a chloramphenicol resistance cassette (promoter-*cat*-terminator), PCR-amplified from plasmid pTRK669 (101) using primers pTRK669_Cm.F and pTRK669_Cm.R (Table S6). Plasmid pTRK1278 was kindly provided by Meichen Pan (41). A similar plasmid, pBC1-*cat* (pTRK1400), was constructed by replacing the erythromycin resistance cassette in the parental vector used for pTRK1278 with a chloramphenicol resistance cassette (promoter-*cat*-terminator) amplified from pNCC293-*cat* using primers RR_68 and RR_45 (Table S6). All three plasmids (pTRK1401, pTRK1278, and pTRK1400) were tested for transformation efficiency in six commercial *B. lactis* strains (Table S5). Transformation efficiency was calculated as log_10_ cfu/µg of DNA. Each transformation was performed in triplicate (*n*= 3) per strain. Statistical analysis was conducted using two-way ANOVA with α = 0.05.

### Construction of CRISPR-based screening vectors

To construct CRISPR-based screening vectors, components of the native Type I-G CRISPR-Cas system in *B. lactis* Bl-04 were used, including the leader sequence, consensus repeat, the modal native spacer length (35 bp). Target protospacers were identified by scanning the genes of interest for the PAM sequence (5′-TAT-3′), as described previously (41). Spacer sequences were checked against the *B. lactis* Bl-04 genome to avoid off-target effects. Guide RNAs (crRNAs) were generated by assembling the leader sequence with synthetic artificial CRISPR arrays (duplex oligonucleotides; IDT, Coralville, IA, USA) using Splicing by Overlap Extension (SOE) PCR. Each crRNA included the native leader, two consensus repeats flanking a 35 bp spacer, and the BBa_B1006 Rho-independent terminator (iGEM Registry). crRNAs were then assembled in the PciI-digested pBC1-*cat* (pTRK1400) (NEB, Ipswich, MA, USA; Cat# R0655S) using NEBuilder HiFi Assembly Master Mix (NEB, Ipswich, MA, USA; Cat# E2621S), generating the CRISPR-screening vectors (Table S5). Genome cleavage by these vectors either enriched pre-existing natural mutants or caused cell death. For each target gene, three unique protospacers were selected. Thus, three screening vectors were tested per knockout.

### Construction of CRISPR-based editing vectors

Editing templates containing homology arms flanking the intended deletion site were synthesized as gene fragments (Genewiz from Azenta Life Sciences, South Plainfield, NJ, USA) and PCR-amplified with primers that added overlaps for assembly (Table S6). The editing templates were then cloned into BspHI-digested screening vectors (NEB, Ipswich, MA, USA; Cat# R0517S) using NEBuilder HiFi Assembly Master Mix (NEB, Ipswich, MA, USA; Cat# E2621S), yielding the final CRISPR-editing vectors (Table S5). For each target gene, two editing template lengths were tested: short (600 bp flanking homology arms; 1.2 kb total) and long (1,000 bp flanking homology arms; 2 kb total). This design generated a total of six editing vectors per target gene (three spacers × two editing template sizes).

### Plasmid curing

To eliminate the CRISPR-editing plasmids, plasmid curing was initiated from one edited strain for each spacer and editing template combination used in each knockout. Each strain was serially re-streaked on non-selective MRSC agar (no antibiotics) for approximately 10 generations. After each passage, six single colonies were screened for plasmid loss by transfer onto MRSC agar supplemented with chloramphenicol (Cm, 5 μg/mL). Colonies that failed to grow on selective medium were further tested by colony PCR with primers RR_187 and RR_189 (Table S6), and the absence of the plasmid was confirmed by agarose gel electrophoresis.

### Knockout phenotypic analysis

The API 50 CHL assay (bioMérieux, Marcy-l'Étoile, France) was used to assess the utilization of 50 different carbohydrates. Overnight cultures were centrifuged (7,000 rpm, 15 min, 4°C), and the cell pellets were washed twice with PBS before being resuspended in API 50 CHL medium (bioMérieux, Marcy-l'Étoile, France; Cat# 50410) to a turbidity of 2 McFarland. API 50 CH test strips (bioMérieux, Marcy-l'Étoile, France; Cat# 50300) were inoculated with the cell suspension, and sterile mineral oil was added to each well to maintain anaerobic conditions. The trays were incubated at 37°C under anaerobic conditions (80% N₂, 10% CO₂, 10% H₂), and results were recorded at 24 and 48 h. Fermentation was assessed based on color change according to the manufacturer’s instructions, with purple indicating no fermentation and yellow indicating strong acid production. For esculin, a positive result was indicated by blackening of the medium. The assay was performed in three biological replicates (*n*= 3) by using three different strips per strain.

To assess utilization of specific carbohydrates, growth curves were also performed using raffinose and isomaltose as sole carbon sources. Overnight cultures of *B. lactis* Bl-04 knockout strains and the wild-type were centrifuged and washed twice with PBS, following the same procedure as for the API 50 CHL assay. After washing, cell suspensions were adjusted to OD_600_ of 0.6, and a 1% (vol/vol) inoculum of each normalized suspension was used to inoculate modified MRSC medium containing either 2% (wt/vol) raffinose (D-(+)-raffinose pentahydrate; Sigma-Aldrich, St. Louis, MO, USA) or 2% (wt/vol) isomaltose (D-isomaltose; LimeFav, Qingdao, China) as the sole carbon source. As a control, each strain was also grown in modified MRSC medium containing 2% (wt/vol) glucose (D-glucose; Fisher, Pittsburgh, PA, USA). The modified MRSC medium consisted of: 1% (wt/vol) peptone protease No3 (Becton Dickinson, Franklin Lakes, NJ, USA), 1% (wt/vol) beef extract (Gibco, Waltham, MA, USA), 0.5% (wt/vol) yeast extract (Becton Dickinson, Franklin Lakes, NJ, USA), 0.1% (wt/vol) polysorbate 80 (Becton Dickinson, Franklin Lakes, NJ, USA), 0.2% (wt/vol) ammonium citrate (Fisher, Pittsburgh, PA, USA), 0.5% (wt/vol) sodium acetate (Fisher, Pittsburgh, PA, USA), 0.01% (wt/vol) magnesium sulfate (Fisher, Pittsburgh, PA, USA), 0.005% (wt/vol) manganese sulfate (Fisher, Pittsburgh, PA, USA), 0.2% (wt/vol) dipotassium phosphate (Fisher, Pittsburgh, PA, USA), 2% (wt/vol) of the appropriate sugar, and supplemented with 0.05% (wt/vol) L-cysteine- HCl (Sigma-Aldrich, St. Louis, MO, USA). The medium was filter-sterilized using a 0.2 μm filter prior to use. Absorbance OD_600_ was recorded every 30 min for 44 h at 37°C under anaerobic conditions (80% N2, 10% CO2, 10% H2), using a clear, flat-bottom 96-well plate (Corning, Corning, NY, USA) and Tecan microplate reader (Magellan software v7.3-SP2; Tecan, Männedorf, Switzerland). For each strain and condition, growth was measured in five technical replicates (wells), and the experiment was performed in three independent biological replicates. Growth of knockout strains was compared to the wild-type to evaluate the utilization of raffinose or isomaltose.

## SUPPLEMENTAL MATERIAL

Figure S1 aem.01839-25-s0001.pdf Visualization of CRISPR spacer content and layout across relevant strains.

Figure S2 aem.01839-25-s0002.pdf Additional growth curves for select strains.

Supplemental legends aem.01839-25-s0003.docx Legends for Fig. S1 and S2.

Supplemental tables aem.01839-25-s0004.xlsx Tables S1 to S7.

ASM does not own the copyrights to Supplemental Material that may be linked to, or accessed through, an article. The authors have granted ASM a non-exclusive, world-wide license to publish the Supplemental Material files. Please contact the corresponding author directly for reuse.
