# Supplementary figures and images for: Enhanced editing of Bifidobacterium lactis using the endogenous Type I-G CRISPR-Cas system
Source: Appl Environ Microbiol. 2026 Jan 12;92(2):e01839-25. doi: 10.1128/aem.01839-25 (PMC12915315; doi:10.1128/aem.01839-25)

A

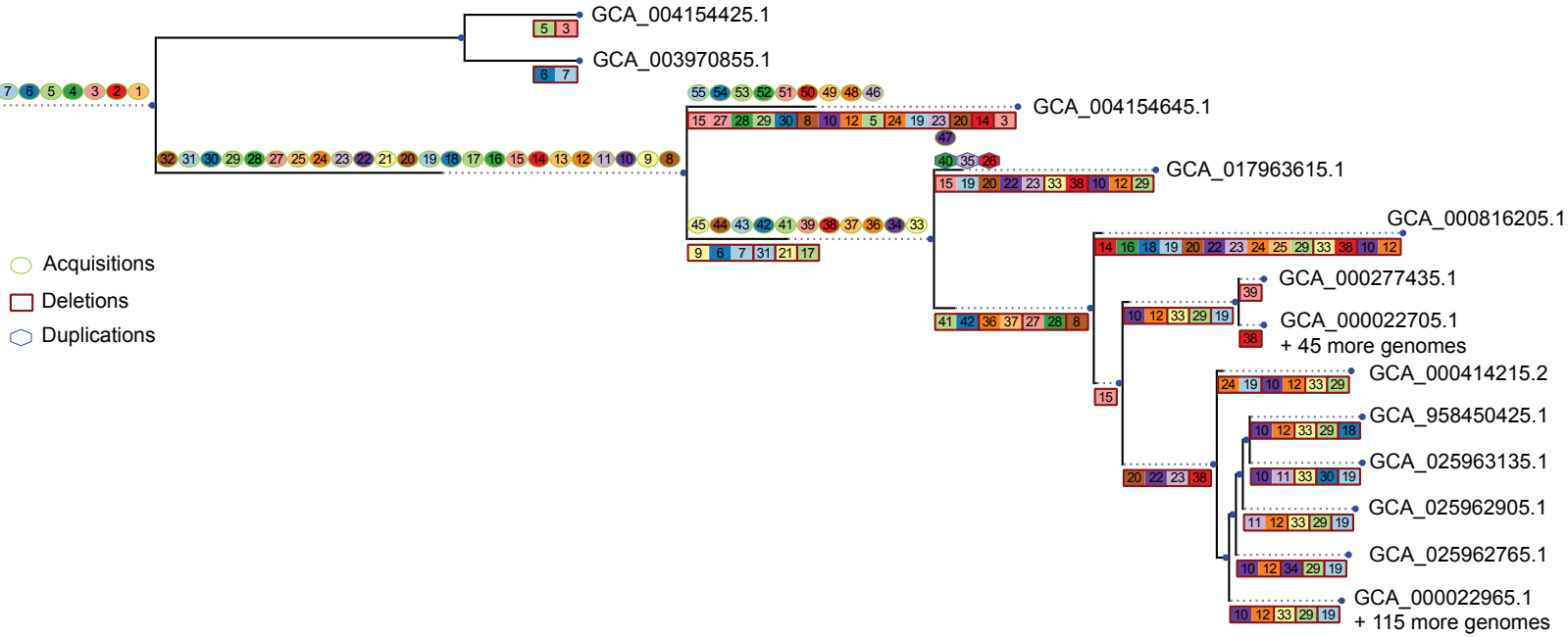

B

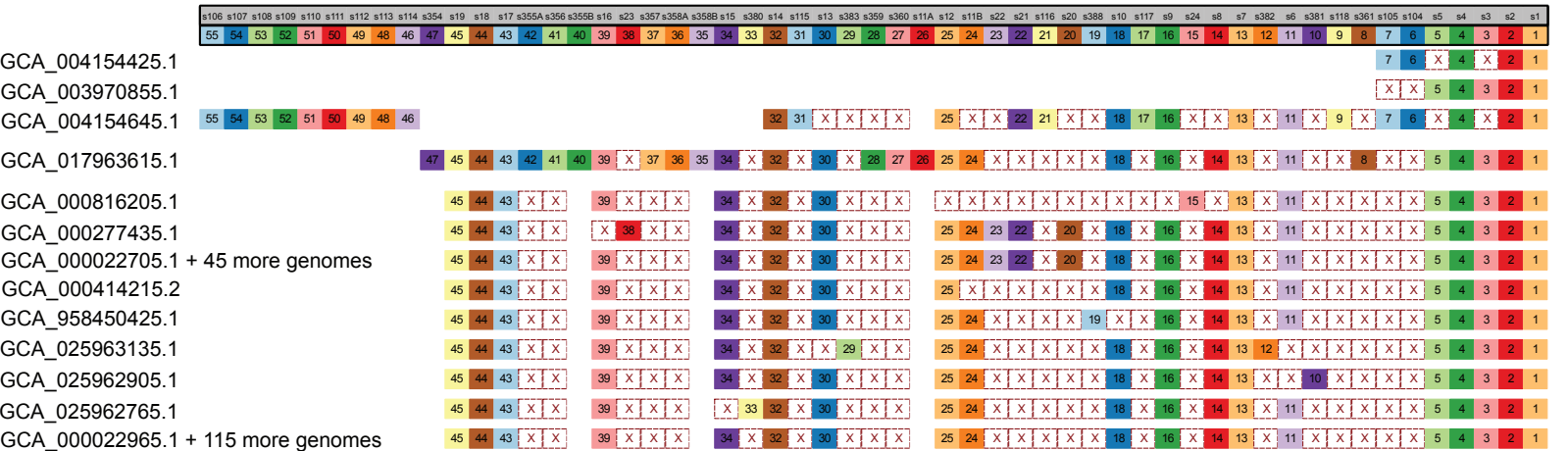

Supplement: Figure S1 — Visualization of CRISPR spacer content and layout across relevant strains. [file aem.01839-25-s0001.pdf]

**A**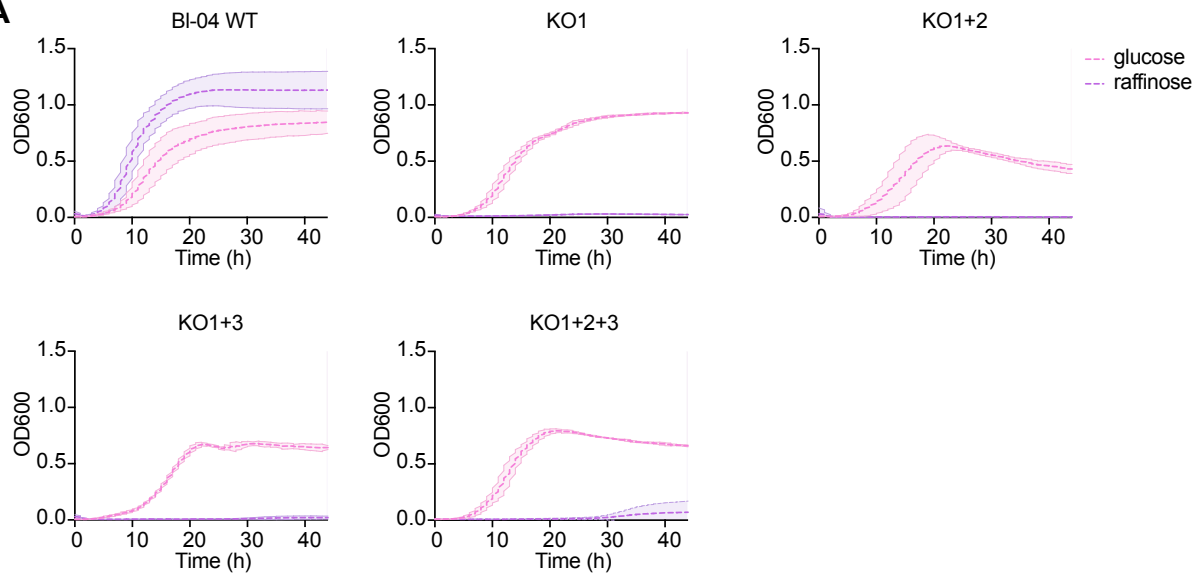**B**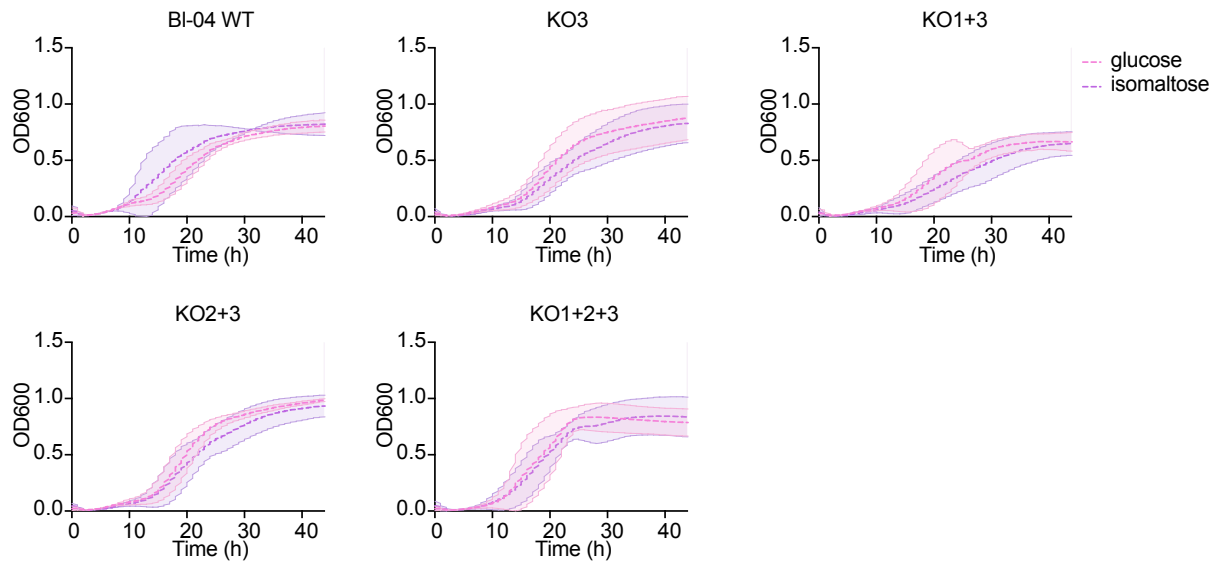

Supplement: Figure S2 — Additional growth curves for select strains. [file aem.01839-25-s0002.pdf]
