# Supplementary material for: Enhanced editing of Bifidobacterium lactis using the endogenous Type I-G CRISPR-Cas system
Source: Appl Environ Microbiol. 2026 Jan 12;92(2):e01839-25. doi: 10.1128/aem.01839-25 (PMC12915315; doi:10.1128/aem.01839-25)
Supplement: Supplemental legends — Legends for Fig. S1 and S2. [file aem.01839-25-s0003.docx]

**SUPPLEMENTAL MATERIAL**

**Figure S1.** Spacer organization dynamics and composition across Type I-G CRISPR arrays in *B. lactis*. (A) Hypothetical reconstruction of CRISPR array evolution based on spacer organization. SpacerPlacer was used to infer putative spacer acquisitions, deletions, and duplications across nonredundant *B. lactis* genomes. Each array configuration is represented by a single genome, and transitions between nodes illustrate predicted spacer-level evolutionary events. (B) Spacer composition profiles for representative Type I-G arrays. Presence-absence patterns of spacer IDs reveal conserved blocks, unique spacers, and variable regions across the arrays. Spacer colors correspond to those in panel A to facilitate comparison between evolutionary trajectories and final array structures.

**Figure S2.** Growth phenotypes of edited isolates on raffinose and isomaltose. **(A)** Growth curves (OD₆₀₀ over time) for wild-type and all edited strains carrying KO1 (KO1, KO1+2, KO1+3, KO1+2+3) in raffinose. **(B)** Growth curves for wild-type and all edited strains carrying KO3 (KO3, KO1+3, KO2+3, KO1+2+3) in isomaltose. Glucose was included as a growth control.
